# PrEP interest and HIV‐1 incidence among MSM and transgender women in coastal Kenya

**DOI:** 10.1002/jia2.25323

**Published:** 2019-06-13

**Authors:** Makobu Kimani, Elise M van der Elst, Oscar Chiro, Clifford Oduor, Elizabeth Wahome, Winston Kazungu, Mahmud Shally, Tobias F Rinke de Wit, Susan M Graham, Don Operario, Eduard J Sanders

**Affiliations:** ^1^ KEMRI‐Wellcome Trust Research Program Kilifi Kenya; ^2^ Amsterdam Institute for Global Health and Development (AIGHD) Department of Global Health University of Amsterdam Amsterdam the Netherlands; ^3^ University of Washington Seattle WA USA; ^4^ Brown University Providence RI USA; ^5^ Nuffield Department of Medicine University of Oxford Oxford UK

**Keywords:** HIV‐1 Incidence, transgender women, TGW, PrEP, men who have sex with men

## Abstract

**Introduction:**

There is emerging data on HIV‐1 incidence among MSM in sub‐Saharan Africa (SSA), but no known estimate of HIV‐1 incidence among transgender women (TGW) in the region has yet been reported. We assessed HIV‐1 incidence and pre‐exposure prophylaxis (PrEP) interest in men who have sex with men exclusively (MSME), men who have sex with men and women (MSMW) and TGW in coastal Kenya.

**Methods:**

HIV‐1‐seronegative individuals who had participated in an HIV testing study in 2016 were traced and retested in 2017 according to Kenyan guidelines. All participants were assigned male sex at birth and had male sex partners; additional data on gender identity and sexual orientation were obtained. We assessed the factors associated with HIV‐1 acquisition using Poisson regression and calculated HIV‐1 incidence in MSME, MSMW and TGW. PrEP interest was assessed through focus group discussions to characterize subcategories’ perceived PrEP needs.

**Results:**

Of the 168 cohort participants, 42 were classified as MSME, 112 as MSMW and 14 as TGW. Overall, HIV‐1 incidence was 5.1 (95% confidence interval (CI): 2.6 to 9.8) per 100 person‐years (PY): 4.5 (95% CI: 1.1 to 17.8] per 100 PY among MSME, 3.4 (95% CI: 1.3 to 9.1) per 100 PY among MSMW and 20.6 (95% CI: 6.6 to 63.8] per 100 PY among TGW. HIV‐1 acquisition was associated with exclusive receptive anal intercourse (aIRR 13.0, 95% CI 1.9 to 88.6), history of an STI in preceding six months (aIRR 10.3, 95% CI 2.2 to 49.4) and separated/divorced marital status (aIRR 8.2 (95%: 1.1 to 62.2). Almost all (98.8%) participants were interested in initiating PrEP. MSME and TGW felt that PrEP would lead to increases in condomless anal or group sex.

**Conclusions:**

TGW had a very high HIV‐1 incidence compared with MSME and MSMW. Subcategories of MSM anticipated different PrEP needs and post‐PrEP risk behaviour. Further studies should assess if TGW may have been wrongly categorized as MSM in other HIV‐1 incidence studies in the region.

## 1 Introduction

Globally, key populations including men who have sex with men (MSM) are at disproportionate risk for HIV‐1 acquisition 1, 2. However, within populations categorized as MSM, there is important variability that carries implications for HIV prevention interventions, such as pre‐exposure prophylaxis. Estimated HIV‐1 incidence in coastal Kenya has been as high as 35.2 (95% confidence interval (CI) 23.8 to 52.1) per 100 person‐years (PY) among MSM who exclusively have sex with men (MSME), compared with 5.8 (95% CI: 4.2 to 7.9) per 100 PY among MSM who have sex with both men and women (MSMW) 1. However, no HIV‐1 incidence has been estimated in transgender women (TGW) in sub‐Saharan Africa (SSA).

A systematic review and meta‐analysis of studies in developed counties reported that TGW have 49 times the odds of having HIV‐1 when compared with the general population 3. The elevated risk for HIV‐1 acquisition in TGW may be due to higher rates of unemployment, drug and alcohol use, transactional sex, homelessness, gender‐based violence and social stigma 4, 5. Due to risk for being re‐victimized by law enforcement, TGW are less likely to report their assaults or go to hospitals for post‐assault care such as post exposure prophylaxis (PEP) 6.

The efficacy of PrEP among high‐risk MSM and TGW has been demonstrated in the iPrEx trial 7, 8. Additional analysis of iPrEX revealed lower PrEP drug concentrations among TGW compared with MSM participants, suggesting problems with adherence among TGW 9.

Since May 2017, Kenya has promoted PrEP use among various at‐risk populations 10. Kenyan PrEP guidelines do not specifically target known risk factors for HIV‐1 acquisition among MSM, including condomless anal intercourse, group sex (i.e. sex with more than one partner during a sexual episode) and the biological sex of sexual partners 11. TGW are not discussed as a population at risk in current Kenyan PrEP guidelines.

As willingness to take PrEP differed among MSMW and MSME in a previous study in Kenya 12, we hypothesized that subgroups of MSM may have different motivations to start PrEP. We further hypothesized that TGW would have different motivations to start PrEP compared with MSM. The aim of this study was to (1) estimate HIV‐1 incidence among different high‐risk subgroups: MSME, MSMW and TGW; and (2) to assess PrEP interest and barriers and facilitators of PrEP adherence among these high‐risk subgroups.

## 2 Methods

### 2.1 Study setting

The study was carried out at the Malindi Sub‐County Hospital in coastal Kenya. Since 2008, KEMRI‐Wellcome Trust Research Project (KWTRP) has been supporting the hospital to provide HIV‐1 testing and counselling to key populations, including MSM, TGW and female sex workers. Engagement with these subgroups was supported by a partnership with *AMKENI*, a community‐based organization serving local key populations.

### 2.2 Recruitment

Inclusion criteria for the cohort study included: male gender at birth, report of a male sex partner in the previous six months, and participation in a parent study of HIV oral self‐testing (OST) conducted between March and June 2016 13. *AMKENI* peer educators were asked to trace all 219 MSM who tested HIV‐1 seronegative in 2016 for retesting with Determine (Abbott, Laboratories, Abbott Park, Illinois, USA), followed by First Response (Premier Medical Corporation, Nadi Daman, India) according to Kenyan national guidelines 14.

Between May and July 2017, prospective participants were screened by study staff who verified previous participation in the OST study, including participant's name (or nickname), age and date of confirmatory HIV‐1 test in 2016. Individuals whose participation in the OST study could not be verified were excluded. All participants underwent HIV‐testing at the enrolment visit. Those who tested HIV‐1 positive were offered ART. The estimated date of infection of these seroconverters was calculated as the mid‐point between their last documented HIV‐1 negative test and the date of study enrolment and repeat testing.

Social‐demographic information including age, education level, marital status and employment status were collected. In addition, participants were asked their gender identity and sexual orientation. Participants were also asked to report risk behaviour over the previous six months, including vaginal intercourse; anal intercourse; role taking during anal intercourse (i.e. insertive, receptive or versatile); receipt of cash, goods or living expenses in exchange for sex, and if they had symptoms suggestive of a sexual transmitted infection (i.e. penile or rectal discharge). TGW were asked to report hormone therapy. All participants did the PrEP interest survey, a 26‐item survey tool capturing knowledge on, and desire to access PrEP, and preferred venue to receive PrEP. For this analysis, MSME was defined when intercourse was reported only with men; MSMW when intercourse was reported with men and women, and TGW when a participant identified as a female.

### 2.3 Focus group discussions

HIV‐1 negative individuals were invited to participate in focus group discussions (FGD). Depending on participant's self‐reported sexual behaviour and gender identity, participants were invited to one of three FGD groups MSME, MSMW and TGW. FDG were facilitated by Kenyan study staff who were fluent in both Kiswahili and English. FGD guides addressed the following general topics: PrEP knowledge, interest to take up PrEP, perceived barriers and facilitators to PrEP uptake and adherence, and preferred PrEP dispensing venue. FGD lasted approximately 90 minutes. Most discussions were conducted in Kiswahili, although English was also used based on participants’ language preference. All discussions were audio‐recorded, transcribed and those conducted in Kiswahili were translated into English. All participants provided written informed consent for FGD. In total, 11 MSMW, 10 MSME and 7 TGW participated in the FGDs.

### 2.4 Data management and analysis

#### 2.4.1 Quantitative analysis

Data from the OST study were used to compare participants in that study who enrolled or did not enrol in the current study. In the 2017 study, data were entered on an online data base (REDCap™ Research Electronic Data Capture). Data cleaning and analysis was done on Stata 15.0 (StataCorp LLC, College Station, Texas, USA). Descriptive statistics were used to compare baseline socio‐demographic and behavioural characteristics of the three subgroups at enrolment. Observation time for each participant was calculated as the time between the HIV‐negative test during the OST study and the date of the current study expressed in terms of PY. HIV‐1 incidence rates were calculated as the number of HIV‐1 incidence cases divided by PY of follow‐up, and expressed as incidence per 100 PY.

We assessed potential predictors of HIV‐1 acquisition using data collected in 2017. Poisson models with robust standard errors were used to obtain population‐averaged incidence rate ratios. Variables significant at *p *≤ 0.2 in bivariable analysis were included in a multivariable model of potential predictors for HIV‐1 acquisition. *p* values were two‐sided and significance was set at *p *≤ 0.05.

#### 2.4.2 Qualitative analysis

Analyses of qualitative data followed the thematic analysis as described by Braun and Clarke 15, which involved systematic coding to identify and define concepts, map the concepts, create typologies, find associations between concepts, and seek explanations from the data. NVivo 10 was used for managing the data. Data were coded by two independent qualitative researchers to ensure that interpretations of quotes were consistent and that data quality was rigorous and transparent; differences between coding were resolved by group discussion involving other members of the research team. Recurring issues, concepts and patterns were identified using both inductive and deductive reasoning. Analyses highlighted whether findings differed by participant subcategories.

#### 2.4.3 Ethics statement

Study procedures were approved by the KEMRI scientific and Ethical Review Unit (KEMRI/SERU/CGMR‐C/0073/3418). All participants provided written informed consent prior to data collection. All participants were informed that PrEP was freely available at Malindi sub‐county hospital.

## 3 Results

Between May and July 2017, 219 MSM participants in the 2016 OST pilot study were targeted for enrolment and of whom 168 (76.7%) enrolled into the 2017 study. The 51 participants who could not be located were more likely than enrolled participants to be MSMW, Muslim and married, and reported more frequent vaginal sex and less frequent receptive anal intercourse in the past six months (data not shown).

Of the 168 enrolled participants, 112 were MSMW, 42 MSME and 14 TGW (Table 1). They had similar background characteristics except for employment status. Overall, the mean age for all participants was 26.7 years (interquartile range: 25.9 to 27.5), 68.5% had primary education only, 83.3% were single and 45.8% were Muslim. Formal employment was higher among TGW compared with MSME and MSMW. Almost all (98.2) had lived in Malindi for two or more years.

**Table 1:** Socio‐demographic and risk perception and PrEP interests of 168 MSM and TGW in Malindi, Kenya, 2016 to 2017

Characteristics	Total (N = 168)	MSMW (N = 112)	MSME (N = 42)	TGW (N = 14)	*p* value
	n (%)	n (%)	n (%)	n (%)	
Age group (years)					
18 to 24	63 (37.5)	38 (33.9)	19 (45.2)	6 (42.9)	0.695
25 to 34	87 (51.8)	62 (55.4)	19 (45.2)	6 (42.9)	
>35	18 (10.7)	12 (10.7)	4 (9.5)	2 (14.3)	
Education					
Primary	115 (68.5)	79 (70.5)	27 (64.3)	9 (64.3)	0.751
Secondary	41 (24.4)	24 (21.4)	13 (31.0)	4 (28.6)	
Higher	12 (7.1)	9 (8.0)	2 (4.8)	1 (7.1)	
Marital status					
Single	140 (83.3)	93 (83.0)	36 (85.7)	11 (78.6)	0.072
Married	14 (8.3)	13 (11.6)	0 (0.0)	1 (7.1)	
Separated/divorced	14 (8.3)	6 (5.4)	6 (14.3)	2 (14.3)	
Religion					
Muslim	77 (45.8)	50 (44.6)	20 (47.6)	7 (50.0)	0.197
Christian	56 (33.3)	33 (29.5)	18 (42.9)	5 (35.7)	
None/other	35 (20.8)	29 (25.9)	4 (9.5)	2 (14.3)	
Employment status					
Employed	29 (17.3)	14 (12.5)	10 (23.8)	5 (35.7)	**0.041** *
Self/un‐employed	139 (82.7)	98 (87.5)	32 (76.2)	9 (64.3)	
Time lived in Malindi					
<2 years	3 (1.8)	1 (0.9)	2 (4.8)	0 (0.0)	0.236
≥2 years	165 (98.2)	111 (99.1)	40 (95.2)	14 (100.0)	
HIV testing frequency last 12 months^a^					
<4 times	128 (76.2)	89 (79.5)	30 (71.4)	9 (64.3)	0.326
≥4 times	28 (16.7)	14 (12.5)	10 (23.8)	4 (28.6)	
Vaginal sex last six months					
Yes	110 (65.5)	107 (95.5)	0 (0.0)	3 (21.4)	**<0.001** ***
Anal sex practice last six months					
IAI only	90 (53.6)	76 (67.9)	12 (28.6)	2 (14.3)	**<0.001** **
RAI only	11 (6.5)	0 (0.0)	4 (9.5)	7 (50.0)	
RAI and IAI	67 (39.9)	36 (32.1)	26 (61.9)	5 (35.7)	
Sexually transmitted infection symptoms in last six months					
Yes	2 (1.2)	0 (0.0)	1 (2.4)	1 (7.1)	**0.048** **
Transactional sex last six months					
Yes	51 (30.4)	27 (24.1)	18 (42.9)	6 (42.9)	**0.042** **
Ever tried getting PEP					
Yes	43 (25.6)	24 (21.4)	14 (33.3)	5 (35.7)	0.213
Ever taken PEP					
Yes	36 (21.4)	19 (17.6)	12 (28.6)	5 (45.5)	0.354
Completed PEP					
Yes	17 (10.1)	11 (10.2)	3 (7.1)	3 (27.3)	0.232
Ever tried getting PrEP					
Yes	4 (2.4)	1 (0.9)	2 (4.8)	1 (9.1)	0.181
Likelihood of using PrEP if offered^b^					
Likely	163 (97.0)	107 (99.1)	42 (100.0)	14 (100.0)	0.631
Not sure	4 (2.4)	4 (3.7)	0 (0.0)	0 (0.0)	
Preferred venue for PrEP access^b^					
Public hospital	28 (16.7)	24 (22.2)	4 (9.5)	0 (0.0)	**0.007**
Private facility	89 (53.0)	64 (59.3)	19 (45.2)	6 (54.5)	
LGBT run community centre	42 (25.0)	20 (18.5)	18 (42.9)	8 (72.7)	
Pharmacy	5 (3.0)	4 (3.7)	1 (2.4)	0 (0.0)	

IAI, insertive anal intercourse; MSME, men having sex with men exclusively; MSMW, men having sex with men and women; RAI, receptive anal intercourse; TGW, transgender women.

^a^Missing 12 values for “HIV testing frequency last 12 Months;” ^b^missing one value for “Likelihood of using PrEP if offered,” “Ever tried to get PrEP” and “Ever tried to get PEP.”

*to denote significance of finding not strong; **indicating stronger significance of the difference; ***Very song significance in the difference seen. Bolded P values indicate that differences between groups were statistically significant (p < 0.05).

Since the OST pilot study in 2016, all participants tested for HIV at least once, with approximately 1 in 4 MSME and TGW taking ≥4 or more HIV tests in the year preceding data collection. There were differences in reported sexual behaviour in the three groups in the past six months: TGW had the highest report of either receptive anal intercourse only, or both receptive and insertive anal intercourse. Two participants (1 MSME and 1 TGW) reported having an STI in the past six months. Approximately one third (32.1%) of all participants reported transactional sex in the past six months, with higher reported transactional sex in MSME (42.9%) and TGW (42.9%) compared with MSM (24.1%). One TGW reported hormonal therapy to support gender transition.

A quarter of all participants had ever tried to get post exposure prophylaxis (PEP); 2.4% had ever tried to access PrEP. Interest to take up PrEP was high across all subcategories. A total of 163 (97%) said that they would take PrEP if it was offered with only four MSMW being unsure. Over half of all participants (53.0%) expressed that the preferred venue to access PrEP would be a private health facility, none of the TGW preferred to collect PrEP from a public hospital, and 42.9% of MSME and 72.7% of TGW preferred to obtain PrEP from a LGBT‐run community centre.

Nine incident HIV‐1 infections occurred: four in MSMW, two in MSME and three in TGW (Table 2). Overall, the estimated HIV‐1 incidence was 5.1 per 100 PY (95% CI: 2.6 to 9.8). Within the subcategories of MSM, HIV‐1 incidence in MSMW was 3.4 per 100 PY (95% CI: 1.1 to 18.2), in MSME 4.5 per 100 PY (95% CI: 1.2 to 9.2) and in TGW 20.6 (95% CI: 6.6 to 63.8) per 100 PY.

**Table 2:** Factors associated with HIV‐1 acquisition among 168 MSM and TGW in Malindi, Kenya, 2017

Characteristics	Incidence/100 PY (95% CI)	Bivariable analysis		Multivariable analysis	
		IRR (95% CI)	*p* value	aIRR (95% CI)	*p* value
All men	5.1 (2.6 to 9.8)				
Subgroup					
MSMW	3.4 (1.3 to 9.1)	Reference		Reference	
MSME	4.5 (1.1 to 17.8)	1.3 (0.3 to 7.0)	0.735	0.8 (0.2 to 3.7)	0.798
TGW	20.6 (6.6 to 63.9)	6.0 (1.5 to 24.2)	0.012	1.5 (0.2 to 10.7)	0.663
Age group (years)					
18 to 24	6.0 (2.2 to 15.9)	Reference		‐	‐
25 to 34	3.3 (1.1 to 10.1)	0.5 (0.1 to 2.4)	0.414		
>35	11.1 (2.8 to 44.4)	1.8 (0.3 to 8.8)	0.498		
Education					
Primary	5.8 (2.7 to 12.1)	Reference		‐	‐
Secondary	4.6 (1.2 to 18.5)	0.8 (0.2 to 3.7)	0.777		
Other	0	‐	‐		
Marital status^a^					
Single	3.4 (1.4 to 8.1)	Reference		Reference	
Married (Heterosexual)	7.2 (1.0 to 51.4)	2.1 (0.2 to 16.0)	0.514	3.9 (0.4 to 38.4)	0.238
Separated/divorced	20.0 (6.5 to 62.1)	4.1 (1.6 to 22.6)	0.008	8.2 (1.1 to 62.2)	0.042
Religion					
Muslim	5.0 (1.6 to 15.6)	Reference		‐	‐
Christian	7.3 (3.3 to 16.2)	1.5 (0.4 to 5.6)	0.586		
None/Other	0	‐	‐		
Employment status^a^					
Formal employment	9.9 (3.2 to 30.7)	Reference		Reference	
Self/un‐employed	4.1 (1.8 to 9.1)	2.4 (0.6 to 9.1)	0.198	3.3 (0.9 to 11.6)	0.061
Time lived in Malindi					
<2 years	0				
≥2 years	5.2 (2.7 to 10.0)	‐	‐		
HIV testing frequency last 12 months					
<4 times	6.0 (3.0 to 12.0)	Reference		‐	‐
≥4 times	3.2 (0.5 to 23.0)	0.6 (0.1 to 4.4)	0.592		
Vaginal sex last six months					
No	6.4 (2.4 to 17.0)	Reference		‐	‐
Yes	4.4 (1.8 to 10.5)	0.7 (0.2 to 2.4)	0.523		
Anal sex practice last six months					
IAI only	4.4 (1.6 to 11.6)	Reference		Reference	
RAI only	17.1 (4.3 to 68.5)	4.1 (0.8 to 19.9)	0.081	13.0 (1.9 to 88.6)	0.009
RAI and IAI	4.1 (1.3 to 12.6)	1.0 (0.2 to 4.4)	0.992	1.4 (0.4 to 5.2)	0.611
Transactional sex last six months					
No	4.1 (1.3 to 9.8)	Reference		‐	
Yes	7.3 (2.7 to 19.5)	1.8 (0.5 to 6.6)	0.351		
History of having a sexually transmitted infection in last six months^a^					
No	4.6 (2.3 to 9.1)	Reference	0.158	Reference	
Yes	53.4 (7.5 to 379.1)	10.4 (2.2 to 48.7)	0.003	10.3 (2.2 to 49.4)	0.003

aIRR, adjusted incidence rate ratio; CI, confidence interval; IAI, insertive anal intercourse; IRR, incidence rate ratio; MSME, men having sex with men exclusively; MSMW, men having sex with men and women; RAI, receptive anal intercourse; TGW, transgender women.

a Only factors significant at *p <* 0.2 in the bivariable model were retained in the multivariable model.

In multivariable analysis, HIV‐1 acquisition was strongly associated with exclusive receptive anal intercourse adjusted (incidence rate ratio (aIRR) 13.0, 95% CI 1.9 to 88.6), history of an STI in preceding six months (aIRR 10.3, 95% CI 2.2 to 49.4), and separated/divorced marital status (aIRR 8.2 (95%: 1.1 to 62.2), while a self‐or unemployed status had a borderline significance (aIRR 3.3 (95%: 0.9 to 11.6, *p* = 0.06) in a model controlling for risk group.

### 3.1 Qualitative findings

A total of 11 MSMW, 12 MSME and 7 TGW (all HIV‐negative) participated in three sub‐group distinct FGDs. Four themes regarding PrEP implementation emerged from the qualitative analysis, revealing some commonalities and distinctions by subgroup regarding their stated interests and concerns related to PrEP (Table 3).

**Table 3:** Summary themes identified from the FGDs with MSME, MSMW TGW

Major themes		Sub‐themes	Representative quote
*PrEP awareness, regardless of sexual orientation*			
1	Information and PrEP literacy	PrEP efficacy knowledge	*PrEP will not protect you from STIs, but I think it is about 99% protective for HIV. I think it is just about the same as Trust (condoms) which are 100% protective (MSMW)*
		Awareness of PrEP procedures	*For seven days, you take it (PrEP) like at 7 in the morning. After seven days when the drug is concentrated enough, you can have sex probably with an infected person and you will be protected, after that you'll continue to take (PrEP) because that is how you will be fully protected (MSME)*
		Requesting information before starting PrEP	*….I would first prefer to get proper information about its side effects. You know…maybe the PrEP drugs require that I take it in on a full stomach, yet I'm a hustler (of low economic status) (TGW)*
2	Consultation about PrEP	Self‐consultation	*…. the first person to consult should be your own self, your inner self, you must ask yourself, do I really need to use this drug?…(MSMW)*
		Peer consultation	*One should consult his peers, whom they identify with. I think consulting them would bring more sense than consulting a person who has no clue about your sexual orientation… (TWG)*
3	Awareness of risk compensation		*The fact is, most of us… Trans, will stop to use condoms, upon starting PrEP (TGW) Group sex will increase, people will have the mentality that we will not get HIV so people will be rough and they will not use protection …(MSME)*
*Barriers to PrEP uptake considered specific to MSM subcategories*			
1	HIV‐related stigma	Anticipatory stigma	*The problems can arise if the drugs (PrEP) are seen in public…because someone I know might be at that place (where the drugs are seen) and then they will go tell people that I have AIDS. How can I even explain to them that these are not ARVs? (MSMW)*
		Homophobic context	*You see, we will be branded sinners … at the hospitals… the kind of people we are… (MSME)*
2	Daily dosing regimen	Uncertainty of daily adherence	*What is boring about this (PrEP), is the daily … like there is a friend of mine who was very excited when he heard about it, but when he realized one has to take it daily, he said: “If this is the case then I will never use it” (MSMW)*
	
3	Fear for side effects		*The fact that one has to take it (PrEP) daily my feeling was that it may destroy the kidneys, rather I would have HIV. So, how to protect myself from HIV without getting kidney failure? (MSMW)*
4	Concomitant drug use	Forgetfulness/interruption	*Sure, alcohol can make one to forget taking his pills* (MSMW)
*Motivations to embark on PrEP*			
1	Sense of relief	Peace of mind	*When I heard about PrEP, I was very pleased by it [ilinipunga] because I want to live well without any worries. (MSME)*
		Opportunity for increased income	*Individuals like us who do sex work, can benefit most. This is because some of us don't use condoms. Therefore, PrEP can guarantee an individual of maximum protection against HIV during an unprotected sexual encounter with an HIV infected person (TWG)*
		More pleasurable (condomless) sex	*I also fear condom breaks, but I also do not like using condoms during sex. I like having unprotected sex. This is what is more pleasurable. Condoms reduce the pleasure. If I had a choice, yes, I would rather not use condoms (MSMW)*
		Ascribing significance to PrEP being a Trans	*Those playing top (inserters) have no issues because they have a choice to put on a condom and protect themselves, of which is not the case with us. When I go out, I become a strict bottom. So, it's up to me, to take precautionary measure (TGW)*
*Preferred PrEP dispensing location*			
1	Public run versus MSM community run healthcare facilities	MSM specific section	*At the Government hospital, GBMSM are not free to be themselves. Sometimes there is discrimination. But if there was a special place, like XX that is only for GBMSM that would be best (MSMW)*

MSME, men who have sex with men exclusively; MSMW, men who have sex with men and women; TGW, transgender women.

### 3.2 PrEP awareness and potential for risk compensation

Participants in all subcategories expressed knowledge on PrEP including efficacy and mode of action, as exemplified by a member of the MSME group (Quote A, Table 3) Additionally, the limitations of PrEP were noted across all subgroups, as noted by a member of the MSMW group who acknowledged that *PrEP will not protect you from STIs*.

However, we observed subgroup differences regarding acknowledgment about the possibility of increased sexual risk behaviour following PrEP uptake (i.e. risk compensation), particularly alluding to erratic condom use. Members of the TGW and MSME group were especially likely to acknowledge the potential reduction in condom use (Quotes B and C, Table 3).

### 3.3 Barriers to PrEP uptake

All subcategories commented on the possibility for HIV‐related stigma due to PrEP, and feared that PrEP medication would be confused by others in their social networks (e.g. family members, partners) with anti‐retroviral drugs, as one member of the TGW group noted *the pill that looks like the medication for HIV‐positive patients*. MSME particularly talked about anticipated enacted stigma in the context of their homophobic social environment, and expressed fear that healthcare providers may not be willing to offer them PrEP (Quote D, Table 3).

Across the subgroups, we observed concerns about the potential need to disclose to partners or family members about the reasons for taking PrEP medications. Participants across subgroups described the dilemma of living a “double life” such that their partners or family members were unaware of their sexuality or gender identity. Participants from both MSM subgroups discussed the likelihood that *promiscuity would be blamed on them….*. As one TGW remarked: *What explanation will a Trans like me, who has a wife and family, give?* Another TGW expressed concern that being witnessed using PrEP would lead others to mistake them as sex workers:

…. but it can be very challenging at times, especially to us who are not sex workers. I'm not a sex worker; I'm in a steady relationship…Yes, PrEP is a new good thing, but convincing your faithful partner that it protects against HIV………. it will raise suspicion. It can be much easier to a sex worker… but it may not be applicable to a Trans who has a faithful partner……(TGW)

In addition, participants commented on PrEP adherence challenges. Daily dosing was expressed as a barrier to PrEP, especially noted by those in the MSMW group (Quote E, Table 3). Other barriers to PrEP adherence were noted by TGW participants, including the likelihood for missed doses due to alcohol or drugs, perceived risk for interactions between PrEP and hormones, and potential side effects.

### 3.4 Motivations to initiate PrEP

PrEP availability was described in all subgroups as a welcome “relief” and PrEP information helped them in getting answers to their questions.

MSME and TGW in particular described that receptive condomless sex for them was common and often a consequence of alcohol and or drug use. As such, members of these groups felt that *PrEP could be an alternative, or extra ‘layer’ of protection*. TGW also noted that PrEP could help to protect against inadvertent disclosure of their “double life.” As a TGW remarked on the potential for PrEP to protect female partners from HIV transmission: *Yes, I'm married to a lady, but at the same time, I identify myself as a lady. Therefore, I secretly have a sexual relationship with a man, because I feel I'm a woman… (With PrEP) I will be able to protect my wife and family, while at the same time fulfilling my sexual desires by going out as a lady*.

### 3.5 Preferred PrEP dispensing location

Across groups, participants preferred PrEP to be dispensed either at LGBT operated clinics or private health facilities (Quote F, Table 3). Despite the government's endorsement of PrEP availability, participants in all three subcategories felt PrEP is still a controversial and divisive issue among health staff in general health clinics Kenya, hence they did not *perceive them as suitable to dispense PrEP to MSM*.

## 4 Discussion

This study characterizes potential differences in HIV risk and PrEP interest between subgroups that are typically categorized as MSM in Kenya—MSME, MSMW and TGW. Through disaggregating subgroups, we found a very high HIV‐1 incidence in TGW, in comparison with incidence estimates for MSMW and MSME. It is possible that an earlier HIV‐1 incidence estimate in MSME of 35.2 per 100 PY in coastal Kenya may have included TGW 1. In the present study, TGW and MSME reported transactional sex more frequently than MSMW, and TGW had the highest reports of receptive anal course. While our study had few seroconverters, participants who reported only taking the receptive role during anal sex, who were separated or divorced, who had a history of a sexually transmitted infection, or who were unemployed or self‐employed had an increased risk of HIV‐1 acquisition. The high incidence in TGW is likely indicative of unmet prevention needs 3.

Globally, TGW have been underserved and have shown to have an exceptionally high HIV‐1 burden 3, 16, 17. Because TGW identify as female, they may prefer not to be identified alongside MSM 18. The recent formation of a Kenyan community‐based organization exclusively for transgender individuals may attest to the preference for specific services for TGW.

In this study, interest to PrEP was high in all three groups studied, suggesting that participants were sensitized about PrEP by peer educators prior to study start. In contrast to findings from a systematic review that indicated that less than a third of MSM in low‐ and middle‐income countries were unaware of PrEP 19.

Motivations to start PrEP varied by subgroup. While PrEP's effectiveness in conjunction with condoms was mentioned, MSME and TGW expressed particularly strong interests in PrEP in comparison with MSMW. Relatively little is known about risk compensation after PrEP initiation outside of trial settings 20. MSME and TGW participants commented that they may increase their risk taking behaviour, and that they were also unsure about taking PrEP daily, whereas these comments were not raised by MSMW participants 21. Although community concerns about possible interactions between PrEP and feminizing hormones have been noted in previous research 22, participants in this study did not note such a concern.

Participants in all subgroups expressed strong disapproval of government hospitals as the venues for dispensing PrEP. This sentiment may reflect the limited skills training among healthcare to work effectively with MSM and TGW patients 23, and also suggests that national prevention programmes in Kenya do not yet note specific considerations for PrEP implementation with MSM or TGW populations 11.

There are limitations to this research. First, the sample of TGW was small, as recruitment depended on participants’ willingness to disclose their gender identity. Second, the data were obtained from a convenience sample recruited through an LGBT community based organization. Third, because participants had not actually used PrEP, discussions reflected hypothetical concerns. Lastly, the study was conducted around the time of national PrEP rollout, which may have influenced participants’ PrEP interest and knowledge.

## 5 Conclusions

There is variability within the population categorized as MSM that has implications for HIV incidence estimates and HIV prevention interventions, including PrEP. TGW in the Kenyan coast represents a previously unresearched group, and has not yet been targeted in HIV prevention programming in Kenya. As TGW have among the highest HIV‐1‐ acquisition risks empirically documented in Kenya, they would benefit from specific PrEP adherence support. Further research on PrEP and other HIV prevention strategies with MSME, MSMW and in particular with TGW is needed to identify specific public health promotion models that maximally respond to the specific needs of these unique vulnerable at‐risk populations.

## Competing interests

The authors declare no conflict of interest.

## Authors’ contributions

EJS, EMvdE and MK conceived and designed the study, while the parent study of oral self‐testing had been done by EMvdE. MK coordinated the study. WK and MS consented participants and did the HIV‐1 testing and counselling. OC provided logistical support for study activities. CO managed the database for the quantitative data. MK transcribed the audio recordings. EW supported MK with quantitative data analysis. EMvdE independently reviewed the coding of the transcripts. EJS provided overall oversight for fieldwork and supervision. TFRdW, SMG and DO supported the manuscript writing and provided oversight. All authors critically reviewed and approved of the write‐up.
